# Worm Proteins of *Schistosoma mansoni* Reduce the Severity of Experimental Chronic Colitis in Mice by Suppressing Colonic Proinflammatory Immune Responses

**DOI:** 10.1371/journal.pone.0110002

**Published:** 2014-10-14

**Authors:** Marthe Heylen, Nathalie E. Ruyssers, Joris G. De Man, Jean-Pierre Timmermans, Paul A. Pelckmans, Tom G. Moreels, Benedicte Y. De Winter

**Affiliations:** 1 Laboratory of Experimental Medicine and Pediatrics, Division of Gastroenterology, University of Antwerp, Antwerp, Belgium; 2 Laboratory of Cell Biology and Histology, Department of Veterinary Sciences, University of Antwerp, Antwerp, Belgium; 3 Antwerp University Hospital, Division of Gastroenterology & Hepatology, Antwerp, Belgium; Institut Pasteur de Lille, France

## Abstract

Although helminthic therapy as a possible new option to treat inflammatory bowel disease is a well-established concept by now, the search for immunomodulatory helminth-derived compounds and their mechanisms of action is still ongoing. We investigated the therapeutic potential and the underlying immunological mechanisms of *Schistosoma mansoni* soluble worm proteins (*Sm*SWP) in an adoptive T cell transfer mouse model of chronic colitis. Both a curative and a preventive treatment protocol were included in this study. The curative administration of *Sm*SWP (started when colitis was established), resulted in a significant improvement of the clinical disease score, colonoscopy, macroscopic and microscopic inflammation score, colon length and myeloperoxidase activity. The therapeutic potential of the preventive *Sm*SWP treatment (started before colitis was established), was less pronounced compared with the curative *Sm*SWP treatment but still resulted in an improved clinical disease score, body weight loss, colon length and microscopic inflammation score. Both the curative and preventive *Sm*SWP treatment downregulated the mRNA expression of the proinflammatory cytokines IFN-γ and IL-17A and upregulated the mRNA expression of the anti-inflammatory cytokine IL-4 in the colon at the end of the experiment. This colonic immunomodulatory effect of *Sm*SWP could not be confirmed at the protein level. Moreover, the effect of *Sm*SWP appeared to be a local colonic phenomenon, since the flow cytometric T cell characterization of the mesenteric lymph nodes and the cytokine measurements in the serum did not reveal any effect of *Sm*SWP treatment. In conclusion, *Sm*SWP treatment reduced the severity of colitis in the adoptive transfer mouse model via the suppression of proinflammatory cytokines and the induction of an anti-inflammatory response in the colon.

## Introduction

Inflammatory bowel disease (IBD) represents a group of chronic inflammatory diseases of the gastrointestinal tract and primarily includes ulcerative colitis and Crohn’s disease. IBD is characterized by remitting and relapsing episodes of intestinal inflammation that can affect the entire gastrointestinal tract (Crohn’s disease) or remain restricted to the colon (ulcerative colitis) [1]. Although the exact etiology of IBD remains unknown, it has been postulated that intestinal barrier dysfunction and an excessive immune activation result from a complex interaction between environmental factors, genetic predisposition and the gut microbiota [2]–[5]. Luminal antigens breaking through the disturbed intestinal epithelial barrier initiate a cascade of inflammatory responses, which can result in excessive and damaging proinflammatory T helper (Th) 1 and Th17 responses, which, in turn, might overwhelm the control mechanisms of regulatory T (Treg) cells [6], [7]. In healthy conditions, Treg cells play an important role in controlling immune homeostasis and maintaining tolerance against self-antigens [8]. This imbalance in the intestinal immunity of IBD patients, shifting towards the proinflammatory side, leads to intestinal inflammation. Finding ways to influence these immunological processes during intestinal inflammation might contribute to new therapeutic options in IBD. This is why the use of helminths, which are known to exert strong influences on the host’s immune system, is being tested extensively as a novel potential treatment strategy for IBD [9]–[11]. The immune response of the host to protect itself against helminth colonization is generally characterized by a Th2 response, whereas the helminth per se is capable of inducing Treg responses to ensure survival within the host [12]–[14]. Based on these observations, it has been hypothesized that helminths can skew the intestinal immune balance in IBD patients towards a more immunosuppressive state through the induction of Th2 and Treg responses, suppressing the damaging proinflammatory Th1 and Th17 responses and thereby suppressing intestinal inflammation [15].

Helminthic therapy is a well-established concept by now as evidenced by the large number of experimental animal studies and clinical trials that have already been conducted or are currently running. Most of the animal studies or trials performed so far have tested living helminth infection as a treatment in animal models with colitis or in small groups of IBD patients, showing promising results [11], [16]–[21]. More recently, the focus of attention has shifted to the identification of helminth-derived molecules with anti-inflammatory effects because these molecules might overcome potential drawbacks of living helminthic therapy with respect to safety issues and the large scale production under good manufacturing practice (GMP) conditions [22]. Taking this into account, we succeeded in demonstrating a therapeutic effect of two helminth-derived products, i.e. *Schistosoma mansoni* soluble worm proteins (*Sm*SWP) and *Ancylostoma caninum* excretory/secretory proteins (*Ac*ES), in an acute experimental colitis model in mice [23]. In spite of the considerable number of studies conducted so far, the exact mechanisms by which helminths or their products provide protection against intestinal inflammation is not fully understood to date [24].

To further unravel the anti-inflammatory properties of helminth treatment and to investigate their long-term effect on experimental colitis, we tested the effect of *Sm*SWP in a chronic colitis model in mice in a preventive and a curative treatment protocol. We thereby focused on the effect of *Sm*SWP treatment on the cells of the adaptive immune system (i.e., T cells) with their corresponding cytokines or cellular markers at the level of the serum, the mesenteric lymph nodes (MLN) and the colon.

## Materials and Methods

### Mice

C.B.-17 SCID and BALB/c mice were obtained from Charles River (France) and maintained in individually ventilated cages. All mice were female and 8 to 9 weeks of age at the initiation of the experiments.

### Chronic adoptive T cell transfer colitis model

Colitis was induced in immunocompromised SCID mice by the adoptive transfer of CD4^+^CD25^−^CD62L^+^ T cells as described previously [25]. Briefly, CD4^+^CD25^−^CD62L^+^ T cells were isolated from the spleens of BALB/c donor mice using a magnetic CD4^+^CD62L^+^ T cell isolation kit (Miltenyi Biotec GmbH). To induce colitis, 1×10^6^ CD4^+^CD25^−^CD62L^+^ T cells in 100 µl phosphate-buffered saline (PBS) were intraperitoneally (i.p.) transferred into SCID mice. Two to three weeks after this adoptive transfer, SCID mice started to develop colitis. Control mice were i.p. injected with 100 µl PBS and did not develop colitis [25].

### Preparation of helminth mixtures

*Schistosoma mansoni* soluble worm proteins (*Sm*SWP) were always prepared in the same way as described previously [23], [26]. Briefly, 7–8 weeks following infection of hamsters with cercariae (larval stage), adult *Schistosoma mansoni* worms were recovered from the livers by portal venous perfusion. The number of adult worms isolated from the hamsters varied every time. The adult worms were washed and homogenized in a small volume of PBS. The soluble products were extracted by centrifugation from the homogenate. Total protein concentration was determined by the rapid protein-selective colorimetric Bradford method according to the manufacturer’s instructions (Coomassie Plus – The Better Bradford Assay kit, Thermo Scientific) [27], [28]. In brief, bovine serum albumin (BSA) standards with known concentrations were prepared (concentration range 25–1500 µg/ml). Next, blanco, standard and *Sm*SWP samples were pipetted *in duplo* into appropriate microplate wells and the Coomassie plus reagents was added to each well. When the Coomassie dye binds to arginine and lysine residues of proteins, an immediate shift in absorption maximum occurs from 465 nm to 595 nm with a concomitant color change from brown to blue, which was measured spectroscopically with a microplate reader. The standard curve was used to determine the protein concentration of *Sm*SWP samples. *Note:* 500–600 adult *Schistosoma mansoni* worms (males and females) approximately correspond to a *Sm*SWP concentration of 1 mg/ml.

### Experimental design

**Figure 1:**
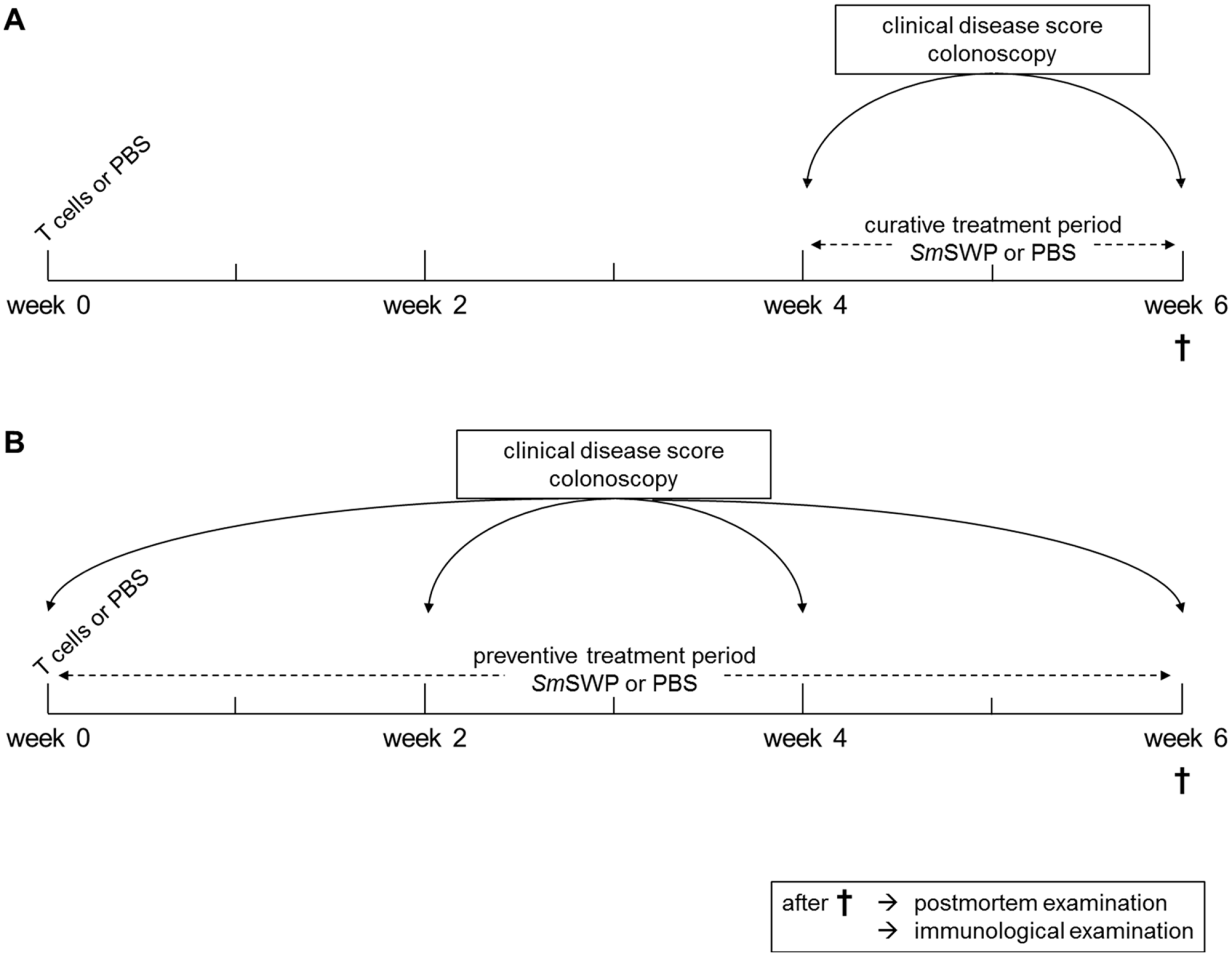
Schematic representation of the experimental design. At week 0, colitis was induced in immunocompromised SCID mice through i.p. injection of CD4^+^CD25^−^CD62L^+^ T cells; controls were injected with PBS. In the curative treatment protocol (**A**), mice were treated with *Sm*SWP or vehicle (PBS) at week 4 and week 5 after T cell or PBS injections (i.e., when colitis was established). In the preventive treatment protocol (**B**), mice were treated weekly for 6 weeks with *Sm*SWP or vehicle (PBS) starting from week 0 (i.e., before colitis was established). During the treatment periods, SCID mice were weighed, a clinical disease score was given and colonoscopy was performed at different time points (weeks 0, 2, 4 and 6). At the end of the study (week 6), mice were sacrificed for postmortem examination of the colon and for examination of the underlying immunological effects of *Sm*SWP. Abbrevations: CD: cluster of differentiation; i.p.: intraperitoneally; PBS: phosphate-buffered saline; SCID: severe combined immunodeficient; *Sm*SWP: *Schistosoma mansoni* soluble worm proteins.

In the curative treatment protocol, SCID mice were treated at week 4 and week 5 after adoptive transfer (i.e., when colitis was established) with vehicle (PBS) or *Sm*SWP at a dose of 25 µg/week (i.p.). In the preventive treatment protocol, SCID mice were treated weekly for 6 weeks with vehicle (PBS) or *Sm*SWP at a dose of 25 µg/week (i.p.) starting from the moment they were adoptively transferred (week 0) (Figure 1A, B). The dose of 25 µg *Sm*SWP in a total volume of 100 µl PBS was chosen based on previous findings from our lab investigating the effect of different doses of *Sm*SWP (5, 10, 25 and 50 µg *Sm*SWP) in a mouse model of acute colitis and showing a maximal effect of 25 µg *Sm*SWP [23]. The time schedule applied (i.e., treatment once a week) was based on Zaccone *et al.* who demonstrated that treatment of non-obese diabetic (NOD) mice with *Sm*SWP once a week completely prevented the onset of type 1 diabetes [23], [29]. The following groups were included in the study: control mice treated with PBS in the preventive and the curative set-up (CONTROL, n = 11), colitis mice treated with PBS in the preventive and the curative set-up (COLITIS, n = 9), colitis mice treated with *Sm*SWP in the curative set-up (COLITIS+cur*Sm*SWP, n = 12) and colitis mice treated with *Sm*SWP in the preventive set-up (COLITIS+prev*Sm*SWP, n = 11).

During the treatment periods, mice were monitored longitudinally via a clinical disease score, their weight progress and endoscopic examinations of the colon [25]. At the end of the study (week 6), mice were sacrificed for postmortem examination of the colon based on 4 parameters (macroscopic and microscopic inflammation score, length of the colon and colonic myeloperoxidase (MPO) activity). To examine the underlying immunological effects of *Sm*SWP, flow cytometric T cell characterization was performed on the cells of the MLN, while the serum of the mice was analyzed via cytometric bead array (CBA) and enzyme-linked immunosorbent assay (ELISA) or real-time polymerase chain reaction (real-time PCR) was performed on the colon supernatants and small pieces of colonic tissue, respectively (Figure 1A, B).

### Clinical examination

At different time points during the treatment periods (at weeks 0, 2, 4 and 6), SCID mice were weighed and a clinical disease score was given to individual mice based on the following parameters: pilo-erection, loose stools and immobility (adapted from Ruyssers *et al.* [23] and Heylen *et al.* [25]). Each parameter was graded from 0 to 2 according to the severity of disease (0 = absent, 1 = moderate, 2 = severe). The cumulative score ranged from 0 to 6.

### Endoscopic examination of the colon

For the continuous monitoring of colitis (at weeks 0, 2, 4 and 6), colonoscopy was performed as described previously using a flexible Olympus URF type P5 ureteroscope with an outer diameter of 3.0 mm and a 1.8 mm working channel (Olympus Europa GmbH) [25]. In brief, mice were anesthetized with a mixture of ketamine (60 mg/kg, Ketalar; Pfizer) and xylazine (6.67 mg/kg, Rompun; Bayer) (i.p.) and placed in a supine position. The anal sphincter and endoscope were lubricated with gel (RMS-Endoscopy) to facilitate insertion of the endoscope. The endoscope was carefully introduced through the anus into the sedated mouse and further inserted as far as possible into the colon under video guidance. Endoscopic scoring was performed during withdrawal of the endoscope. The colonoscopic grading scale was based on the following parameters: colonic translucency, the presence of fibrin attached to the bowel wall, the morphology of the vascular pattern, and the presence of loose stools (scored each between 0 and 3) [25]. The cumulative score ranged from 0 (no signs of inflammation) to 12 (signs of severe inflammation).

### Macroscopic inflammation score and length of the colon

At the end of the experiment (week 6), mice were sacrificed by exsanguination under anesthesia with a mixture of ketamine (90 mg/kg, Ketalar; Pfizer) and xylazine (10 mg/kg, Rompun; Bayer). Colons were removed to score the colonic mucosal damage macroscopically as described previously [25]. The macroscopic inflammation score included the following 4 parameters: ulcerations, hyperemia, bowel wall thickening and mucosal edema. Each parameter was given a score from 0 (normal) to 3 (severe), leading to a cumulative score ranging from a minimum of 0 to a maximum of 12. The length of the colon was also measured and expressed in cm.

### Microscopic inflammation score

Colonic segments, taken in a standardized way, were fixed in 4% formaldehyde, embedded in paraffin and cross sections of 5 µm were stained with hematoxylin-eosin [25]. The following criteria were included in the microscopic scoring system: degree of inflammatory infiltrates in the lamina propria (0–3 points), loss of goblet cells as a marker of mucin depletion (0–1 points), crypt architecture (0–3 points), presence of crypt abscesses (0–1 points), presence of mucosal erosion or ulceration (0–2 points) and layers involved in the inflammation from submucosal toward transmural involvement (0–3 points). The cumulative score ranged from a minimum of 0 to a maximum of 13.

### MPO activity assay

Colonic MPO activity, which is directly related to the number and the activity of neutrophil granulocytes in the inflamed tissue, was assayed according to a previously published method to monitor the degree of inflammation [23], [25], [30]. In brief, a full thickness tissue sample was harvested from the colon in a standardized way. The colonic segment was blotted dry, weighed, and placed in a potassium phosphate buffer (pH 6.0) containing 0.5% hexadecyltrimethylammonium bromide at 5 g of tissue per 100 ml of buffer. The sample was placed on ice, minced, and homogenized for 30 sec. The homogenate was subjected to 2 sonication and freeze-thawing cycles. The suspension was centrifuged at 15000 rpm for 15 min at 4°C. An aliquot (0.1 ml) of the supernatant was added to 2.9 mL of *o*-dianisidine solution (16.7 mg of *o*-dianisidine in 1 ml of methyl alcohol, 98 mL of 50 mM potassium phosphate buffer, pH 6.0, and 1 ml of 0.05% H_2_O_2_ solution as a substrate for the MPO enzyme). The change in absorbance was read at 460 nm over 60 sec with a Spectronic Genesys 5 spectrophotometer (Milton Roy). One unit of MPO activity was defined as the quantity able to convert 1 mmol of H_2_O_2_ to H_2_O per minute at 25°C, and the activity was expressed in units per gram of tissue.

### Mesenteric lymph nodes (MLN): cell isolation and flow cytometric T cell characterization

Single cell suspensions of MLN were prepared as previously described [23]. MLN isolated from mice were mashed through a 40 µm nylon cell strainer (BD Biosciences) using a 1 ml syringe. The cell strainer was rinsed with RPMI 1640 medium and cells were washed in RPMI through centrifugation (1500 rpm for 5 min), after which the cell pellet was suspended in red blood cell lysis buffer (Sigma-Aldrich). Thereafter, cells were washed in RPMI and the cell pellet was suspended in RPMI. Debris was removed from the single cell suspension by washing the cells through a 40 µm nylon cell strainer.

The intracellular cytokine stain was performed on MLN single cell suspensions. Prior to staining, the cells were cultured for 4 h at 37°C and 5% CO_2_ in lymphocyte growth medium (RPMI 1640 medium containing 10% fetal calf serum (FCS), 25 mM HEPES buffer, 2 mM L-glutamine, 50 µM β-mercaptoethanol, 1 mM sodium pyruvate, 1% MEM non-essential amino acids 100x, 2% MEM amino acids 50x, 100 U/ml penicillin and 100 µg/ml streptomycin (all from Life Technologies or Sigma-Aldrich)) containing phorbol myristate acetate (1 µg/ml), ionomycin (1 µg/ml) and brefeldin A (10 µg/ml) (all from Sigma-Aldrich). After 4 h of incubation, the cells were stained with anti-CD4 FITC (clone: RM4-5; BD Biosciences). Then cells were fixed and permeabilized with the BD Cytofix/Cytoperm Fixation/Permeabilization kit (BD Biosciences) and stained with anti-IFN-γ eF450 (clone: XMG1.2; eBioscience), anti-IL-17A PerCP-Cy5.5 (clone: eBio17B7; eBioscience), anti-IL-4 PE (clone: 11B11; BD Biosciences) and anti-IL-10 APC (clone: JES5-16E3; eBioscience).

During the regulatory T cell stain, cells of the MLN were stained with anti-CD4 FITC (clone: RM4-5; BD Biosciences) and anti-CD25 PerCP-Cy5.5 (clone: PC61.5; eBioscience). Then, cells were fixed and permeabilized with the Foxp3 Staining Buffer Set (eBioscience) and stained with anti-Foxp3 APC (clone: FJK-16s; eBioscience).

**Figure 2:**
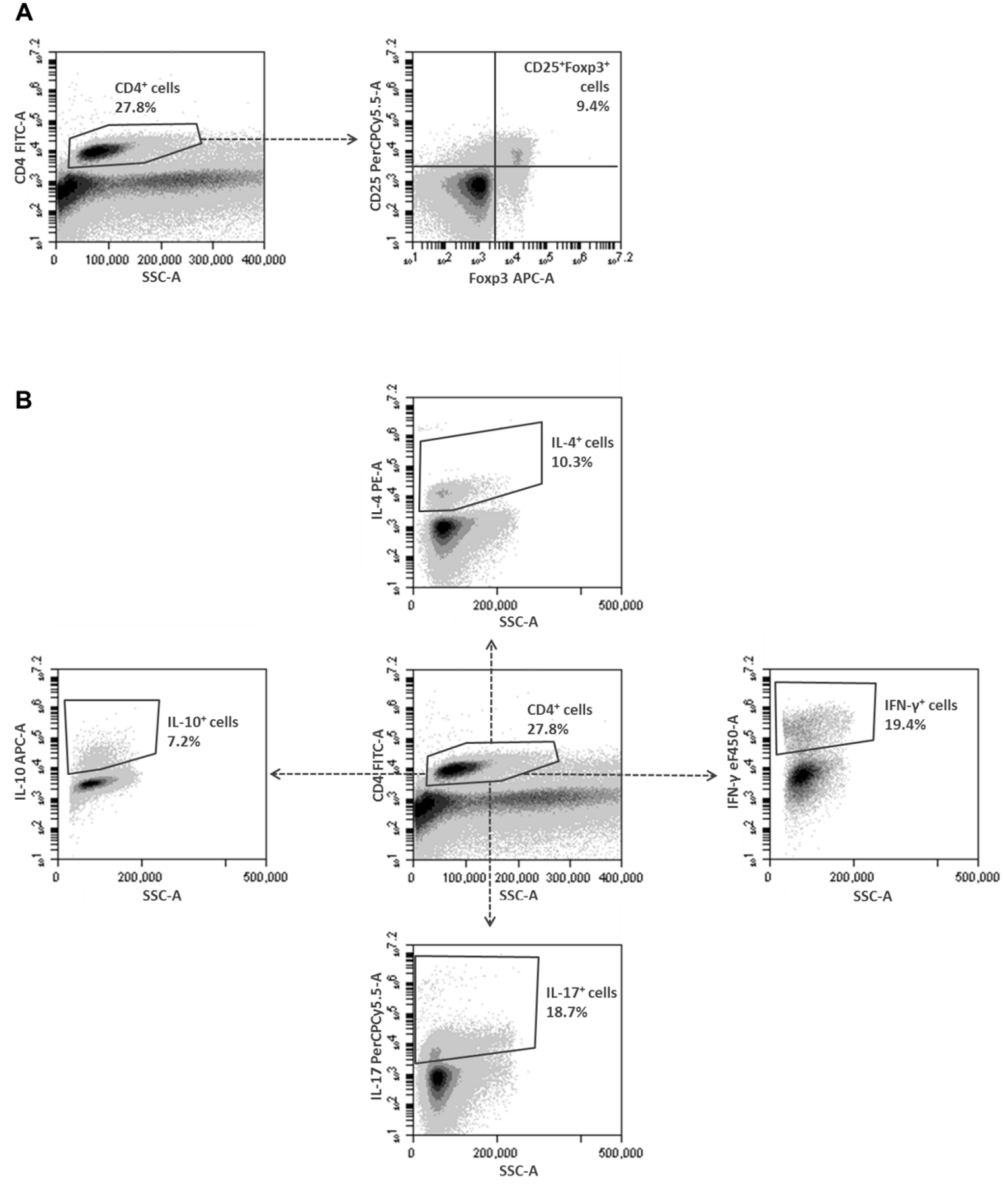
Multi-color flow cytometric gating strategies for the T cell characterization of the MLN cells. Gating strategy for the regulatory T cell stain (**A**): CD4^+^ cells were initially gated upon expression of CD4 and SSC properties, and subsequently gated and assessed upon expression of CD25 and Foxp3. Gating strategy for the intracellular cytokine stain (**B**): CD4^+^ cells were initially gated upon expression of CD4 and side SSC properties, and subsequently gated and assessed upon expression of IFN-γ, IL-17A, IL-4 or IL-10. Abbreviations: CD: cluster of differentiation; Foxp3: forkhead box p3; IFN-γ: interferon-γ; IL: interleukin; MLN: mesenteric lymph nodes; SSC: side scatter.

The cells were examined on a BD FACSAria II (BD Biosciences) and analyzed using FlowJo software (TreeStar). The different cell populations were gated using isotype controls. The gating strategy is shown in Figure 2.

### Colon: cultures and enzyme-linked immunosorbent assay (ELISA)

A full thickness tissue sample was harvested from the colon in a standardized way and incubated for 24 h at 37°C and 5% CO_2_ in complete RPMI 1640 medium containing 10% FCS, 2 mM L-glutamine, 100 U/ml penicillin and 100 µg/ml streptomycin (all from Life Technologies). Colon supernatants were collected after 24 h and stored at −80°C for ELISA analyses.

The colonic levels of IFN-γ, IL-17A, IL-5 and IL-10 were assayed by four-member solid phase sandwich ELISA according to the manufacturer’s instructions (Life Technologies). Blanco, standard, low control, high control and unknown samples were measured *in duplo*. The minimum detectable level was 2 pg/ml for IFN-γ, 5 pg/ml for IL-17A, 3 pg/ml for IL-5 and 13 pg/ml for IL-10. The intra-assay variability (%CV) was 4% for IFN-γ, 4% for IL-17A, 6% for IL-5 and 7% for IL-10. The inter-assay variability (%CV) was 4% for IFN-γ, 4% for IL-17A, 6% for IL-5 and 9% for IL-10.

### Colon: mRNA isolation and real-time polymerase chain reaction (PCR)

A full thickness tissue sample was harvested from the colon in a standardized way, washed and then frozen in liquid nitrogen. Total RNA was extracted from the colonic tissues using a column-based technique (RNeasy Mini Kit, Qiagen). The protocol was adapted from Ledeganck *et al.* [31]. Purified total RNA was treated with DNase to obtain DNA-free RNA (Turbo DNase-free, Life Technologies). cDNA was synthesized using Transcriptor First Strand cDNA Synthesis Kit (Roche Applied Science).

To examine the mRNA expression in the colon of IFN-γ, IL-17A, IL-4 and IL-10, quantitative real-time PCR was performed using the TaqMan Universal PCR Master Mix (Life Technologies). The PCR reaction was performed in a 25 µl reaction volume containing cDNA sample, MasterMix, forward primer, reverse primer and probe. Pre-incubation was performed at 50°C for 2 min and 95°C for 10 min before the PCR cycling (50 cycles) at 95°C for 15 sec (denaturation) and at 60°C for 1 min (annealing/extension). The following primers were used: IFN-γ (gene ID: 15978-Mm01168134_m1; Life Technologies), IL-17A (gene ID: 16171-Mm00439618_m1; Life Technologies), IL-4 (gene ID: 16189-Mm00445259_m1; Life Technologies), IL-10 (gene ID: 16153-Mm00439614_m1; Life Technologies). All genes were normalized against the endogenous housekeeping gene β-actin (gene ID: 11461-Mm00607939_s1; Life Technologies). The fold change of the relative mRNA expression of each studied gene was calculated with the 2^−ΔΔCT^ method [32].

### Blood sampling and cytometric bead array (CBA)

Blood was collected from mice by cardiac puncture and put into SSTII Advance Blood Collection Tubes (BD Vacutainer). After centrifugation (3500 rpm, 20 min), serum was collected and stored at −80°C for CBA.

The levels of IL-2, IL-4, IL-6, IFN-γ, TNF, IL-17A and IL-10 were measured in the serum samples using a CBA Mouse Th1/Th2/Th17 Cytokine Kit according to the manufacturer’s instructions (BD Biosciences). Blanco, standard and unknown samples were measured *in duplo*. The minimum detectable level was: 0.1 pg/ml for IL-2, 0.03 pg/ml for IL-4, 1.4 pg/ml for IL-6, 0.5 pg/ml for IFN-γ, 0.9 pg/ml for TNF, 0.8 pg/ml for IL-17A and 16.8 pg/ml for IL-10. The intra-assay variability (%CV) was 3% for IL-2, 3% for IL-4, 6% for IL-6, 3% for IFN-γ, 4% for TNF, 2% for IL-17A and 11% for IL-10. The inter-assay variability (%CV) was 3% for IL-2, 6% for IL-4, 4% for IL-6, 3% for IFN-γ, 5% for TNF, 4% for IL-17A and 6% for IL-10.

### Presentation of results and statistical analysis

Data are presented as mean ± SEM in different formats (bar graphs, table and dot plots in which each dot represents a separate mouse), for “n” representing the number of mice. The generalized estimating equations model was used to analyze the evolution of the clinical disease score and the colonoscopic score in time and a least significant difference (LSD) post-hoc analysis was applied whenever appropriate. One-way ANOVA with LSD post-hoc test was used to compare the results of macroscopic and microscopic scores, colon length, MPO activity, flow cytometry, ELISA, real-time PCR and CBA between groups. An unpaired Student’s *t* test was performed to compare the flow cytometric and real-time PCR results between the COLITIS and the COLITIS+*Sm*SWP groups. *P* values of ≤0.05 were considered as significant. Data were analyzed using SPSS 18.0 software and GraphPad Prism 5.00.

### Ethics Statement

All animal experiments were performed in strict accordance with the guidelines of the Ethical Committee on Animal Experimentation at the University of Antwerp (Belgium) that approved the study protocol (permit number: 2010–28). Animal experiments were performed by researchers holding a FELASA category C certificate.

## Results

### Effect of curative *Sm*SWP treatment on the inflammatory parameters during colitis

**Figure 3:**
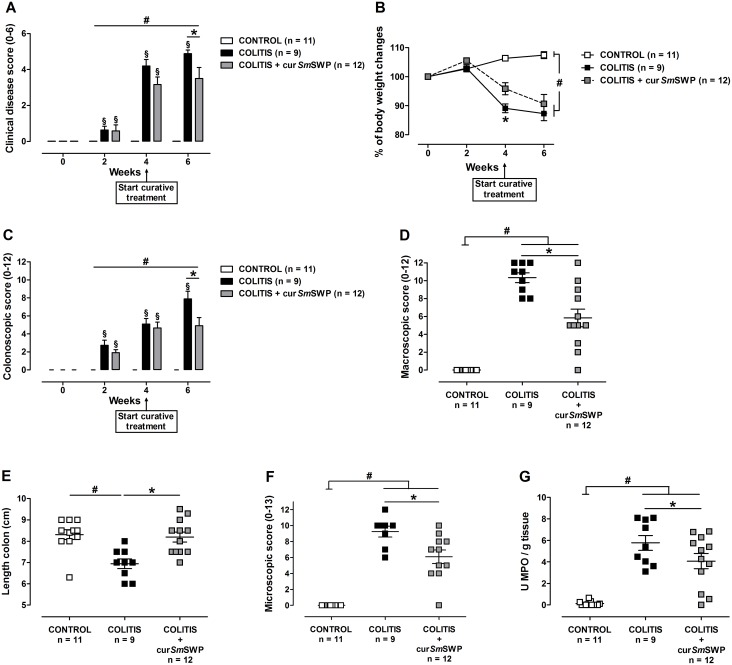
Effect of curative treatment with *Sm*SWP on inflammatory parameters. Effect on clinical disease score (**A**), body weight (**B**), colonoscopic score (**C**), macroscopic inflammation score (**D**), colon length (**E**), microscopic inflammation score (**F**) and MPO activity (**G**). Data are presented as mean ± SEM. Generalized Estimations Equations was used to analyze the evolution of the body weight, the clinical disease score and colonoscopic score over time and an LSD post-hoc analysis was applied. One-way ANOVA with LSD post-hoc test was used to compare the results of macroscopic and microscopic scores, colon length and MPO activity between groups. ^#^: *P*≤0.05, significantly different from CONTROL group; *: *P*≤0.05, significant difference between the COLITIS and COLITIS+cur*Sm*SWP groups; ^§^: *P*≤0.05, significant increase in score between week 4 and week 6 for the COLITIS group; “n” representing the number of mice. Abbreviations: cur: curative; LSD: least significant difference; MPO: myeloperoxidase; SEM: standard error of the mean; *Sm*SWP: *Schistosoma mansoni* soluble worm proteins.

CONTROL mice showed no clinical signs of illness and gained weight during the treatment period (Figure 3A, B). The clinical disease score of COLITIS mice increased significantly over time, whereas the clinical disease score of COLITIS+cur*Sm*SWP mice remained stable during the treatment period (i.e. from week 4 to week 6) and was significantly lower than the score of COLITIS mice at week 6 (Figure 3A). The curative administration of *Sm*SWP had no effect on the body weight, as both COLITIS and COLITIS+cur*Sm*SWP mice lost weight during the treatment period (Figure 3B).

**Figure 4:**
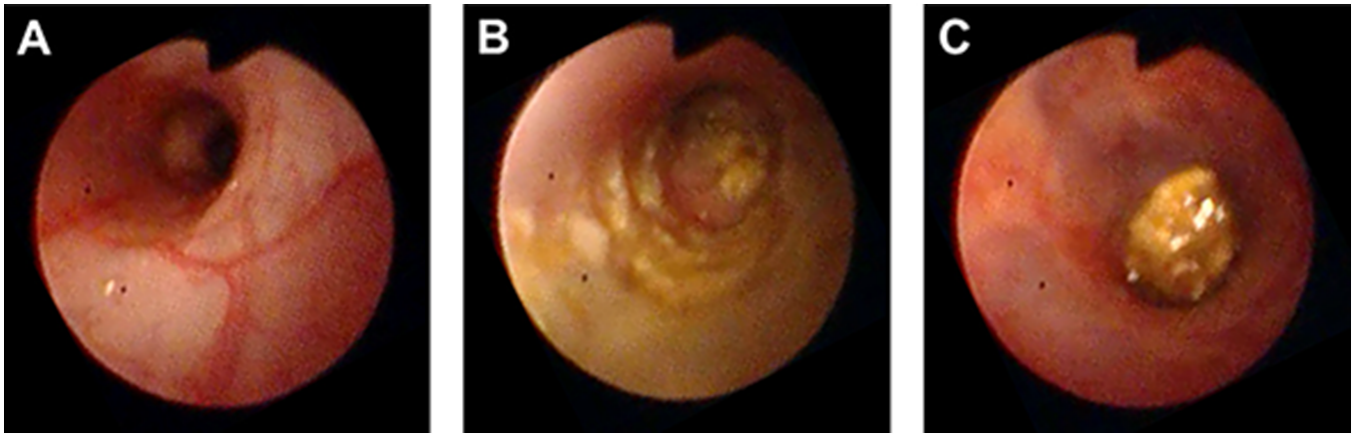
Effect of curative *Sm*SWP treatment on mucosal damage as seen during colonoscopy. Representative pictures of colonoscopy at week 6 for: (**A**), a CONTROL mouse with a normal vascular pattern and smooth transparent mucosa; (**B**), a COLITIS mouse with spread loose stool, thickening of the colon wall and reduced transparency of the mucosa; (**C**), a COLITIS+cur*Sm*SWP mouse with a more transparent mucosa, almost normal branched blood vessel structures and loose but still shaped stool. Abbreviations: cur: curative; *Sm*SWP: *Schistosoma mansoni* soluble worm proteins.

Colonoscopic examination of CONTROL mice showed a thin translucent colonic wall characterized by a smooth and shiny mucosa and normal blood vessel architecture at week 6 (Figure 4A), resulting in a colonoscopic score of 0 (Figure 3C). Colonoscopy of COLITIS mice at the end of the experiment revealed mucosal inflammation characterized by marked changes of the vascular pattern, thickening of the colon wall and loose unshaped stools (Figure 4B). COLITIS+cur*Sm*SWP mice showed reduced signs of mucosal inflammation at week 6 with a more translucent colonic wall, an almost normal branched blood vessel structure and loose but still shaped stools (Figure 4C). The curative administration of *Sm*SWP thus had a positive effect on the mucosal damage of the colon of COLITIS+cur*Sm*SWP mice since the colonoscopic score remained stable between weeks 4 and 6, i.e., the treatment period (4.7±0.7 and 4.9±0.9, respectively) and was significantly lower than the colonoscopic score of COLITIS mice at week 6 (7.9±0.9) (Figure 3C).

The beneficial effect of the curative *Sm*SWP treatment was also confirmed by the macroscopic inflammation score and the microscopic inflammation score, which were significantly lower for COLITIS+cur*Sm*SWP mice compared with COLITIS mice (Figure 3D, F). Furthermore, MPO activity was also significantly lower in COLITIS+cur*Sm*SWP mice compared with COLITIS mice (Figure 3G). CONTROL mice showed no macroscopic or microscopic damage and low colonic MPO activity (Figure 3D, F, G). The colon length of the COLITIS mice significantly decreased compared with CONTROL mice (6.9±0.2 cm and 8.3±0.2 cm, respectively) (Figure 3E). Curative administration of *Sm*SWP significantly increased colon length reaching normal values in the COLITIS+cur*Sm*SWP mice (8.2±0.2 cm) (Figure 3E).

### Effect of curative *Sm*SWP treatment on the immunological response and the immunological profile during colitis

To examine the underlying immunological effects of curative *Sm*SWP treatment, flow cytometric T cell characterization was performed on cells of the MLN, the serum was analyzed by CBA and ELISA and real-time PCR was performed on the colon supernatants and on colon tissue, respectively.

**Figure 5:**
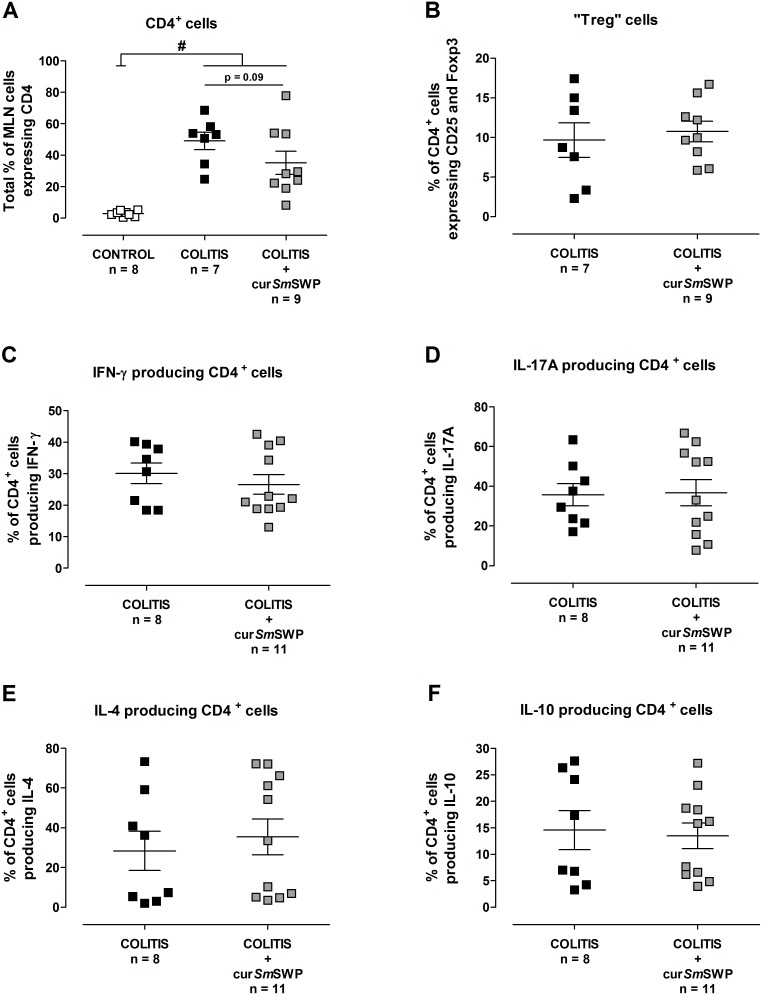
Flow cytometric T cell characterization of the MLN cells after curative *Sm*SWP treatment. Total % of CD4^+^ cells (**A**), % of CD4^+^ cells expressing CD25 and Foxp3 (probably Treg cells) (**B**), % of CD4^+^ cells producing IFN-γ (**C**), % of CD4^+^ cells producing IL-17A (**D**), % of CD4^+^ cells producing IL-4 (**E**) and % of CD4^+^ cells producing IL-10 (**F**). Data are presented as mean ± SEM. One-way ANOVA with LSD post-hoc test or an unpaired Student’s *t* test was used as an appropriate approach to compare the flow cytometric results between groups. ^#^: *P*≤0.05, significantly different from CONTROL group; “n” representing the number of mice. Abbreviations: CD: cluster of differentiation; cur: curative; Foxp3: forkhead box p3; IFN-γ: interferon-γ; IL: interleukin; LSD: least significant difference; MLN: mesenteric lymph nodes; %: percentage; SEM: standard error of the mean; *Sm*SWP: *Schistosoma mansoni* soluble worm proteins.

Flow cytometric T cell characterization of the MLN cells showed an upregulation of CD4^+^ cells in COLITIS mice and COLITIS+cur*Sm*SWP mice compared with CONTROL mice. However, there was a tendency (*P* = 0.09) towards a lower number of CD4^+^ cells after curative *Sm*SWP treatment (Figure 5A). Expectedly, CD4^+^ cells were (nearly) absent in CONTROL mice because SCID mice are immunodeficient (Figure 5A). Therefore, further T cell characterization was only performed for the COLITIS and COLITIS+cur*Sm*SWP mice. Within the population of CD4^+^ cells, comparable percentages of cells expressing CD25 and Foxp3, probably Treg cells, were found in COLITIS and COLITIS+cur*Sm*SWP mice (Figure 5B). Furthermore, *Sm*SWP treatment did not influence the cytokine production within the CD4^+^ cell population since equal numbers of IFN-γ, IL-17A, IL-4 and IL-10 producing cells were found in the COLITIS and COLITIS+cur*Sm*SWP mice by flow cytometry (Figure 5C–F).

**Figure 6:**
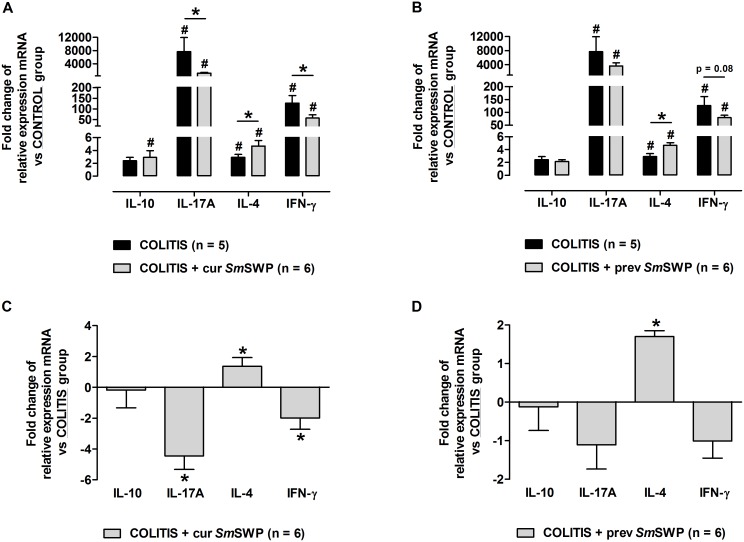
Fold changes of the relative mRNA expression of colonic IL-10, IL-17A, IL-4 and IFN-γ. (**A**) and (**C**) represent the curative set-up of *Sm*SWP treatment, (**B**) and (**D**) represent the preventive set-up of *Sm*SWP treatment. In (**A**) and (**B**), data are expressed as the fold change of relative mRNA expression and the CONTROL group was chosen as calibrator. In (**C**) en (**D**), data are expressed as the fold change of relative mRNA expression and the COLITIS group was chosen as calibrator. Data are presented as mean ± SEM. One-way ANOVA with LSD post-hoc test or an unpaired Student’s *t* test was used as an appropriate approach to compare the real-time PCR data between groups. ^#^: *P*≤0.05, significantly different from CONTROL group; *: *P*≤0.05, significant difference between the COLITIS and COLITIS+*Sm*SWP groups; “n” representing the number of mice. Abbreviations: cur: curative; IFN-γ; interferon-γ, IL: interleukin; LSD: least significant difference; mRNA: messenger ribonucleic acid; PCR: polymerase chain reaction; prev: preventive; SEM: standard error of the mean; *Sm*SWP: *Schistosoma mansoni* soluble worm proteins.

Intestinal inflammation induced by the adoptive transfer of CD4^+^CD25^−^CD62L^+^ T cells in SCID mice significantly augmented the colonic mRNA expression of the proinflammatory cytokines IL-17A and IFN-γ compared with CONTROL mice. The expression of IL-4 and IL-10 mRNA was also increased in the colon of COLITIS mice compared with CONTROL mice, but was less pronounced than the expression of IL-17A and IFN-y mRNA (Figure 6A, B). Curative administration of *Sm*SWP significantly reduced the colonic mRNA expression of IL-17A and IFN-γ (a 4-fold and 2-fold reduction, respectively) and significantly increased the colonic mRNA expression of IL-4 (a 2-fold increase) in COLITIS+cur*Sm*SWP mice compared with COLITIS mice (Figure 6C). No significant difference of the curative *Sm*SWP treatment in the mRNA expression of IL-10 was seen between COLITIS and COLITIS+cur*Sm*SWP mice (Figure 6C).

**Figure 7:**
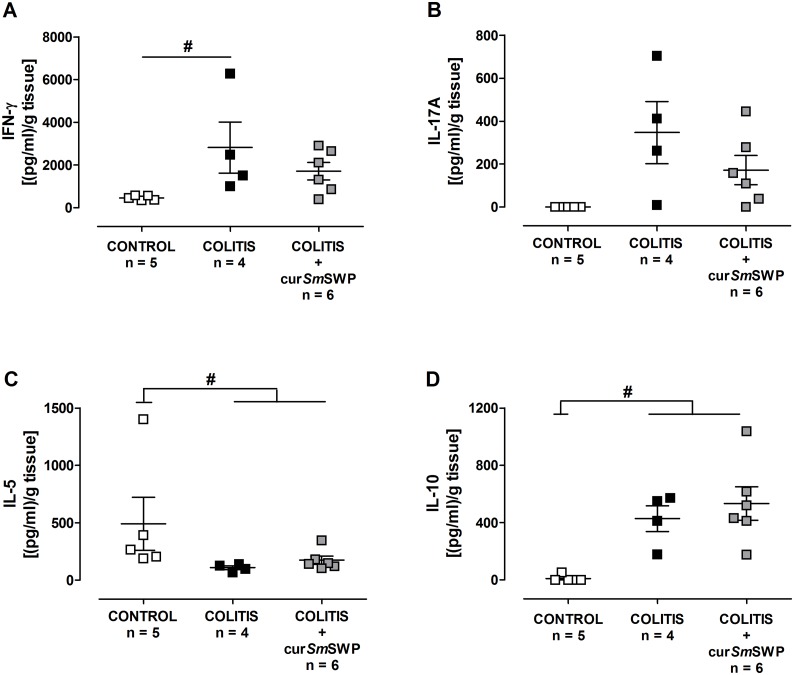
Effect of curative treatment with *Sm*SWP on the cytokine profile in colon supernatants. Concentrations of IFN-γ (**A**), IL-17A (**B**), IL-5 (**C**) and IL-10 (**D**) were measured by ELISA in colon supernatants. Data are presented as mean ± SEM. One-way ANOVA with LSD post-hoc test was used to compare the concentrations between groups. ^#^: *P*≤0.05, significantly different from CONTROL group; “n” representing the number of mice. Abbreviations: cur: curative; ELISA: enzyme-linked immunosorbent assay; IFN-γ; interferon-γ, IL: interleukin; LSD: least significant difference; SEM: standard error of the mean; *Sm*SWP: *Schistosoma mansoni* soluble worm proteins.

Intestinal inflammation in the adoptive transfer colitis model examined by ELISA was also characterized by higher concentrations of IFN-γ, IL-17A and IL-10 in the colon supernatants compared with CONTROL mice (Figure 7). The anti-inflammatory effect of the curative administration of *Sm*SWP as proven by real-time PCR could not be confirmed by ELISA analysis, since no statistical significance was reached between COLITIS and COLITIS+cur*Sm*SWP mice. However, Figure 7 demonstrates that the concentration of the proinflammatory cytokines IFN-γ and IL-17A was lower in the colon supernatants of mice treated with *Sm*SWP in the curative set-up, resulting in a loss of significance between CONTROL and COLITIS+cur*Sm*SWP mice (Figure 7).

CBA analysis showed only low concentrations of the cytokines IL-2, IL-4, IL-6, IFN-γ, TNF, IL-17A and IL-10 in the serum, but revealed differences in the proinflammatory cytokines IL-2, IFN-γ, TNF, IL-6 and IL-17A between CONTROL mice and COLITIS mice (Table 1). Furthermore, no differences in cytokine concentrations were detected between COLITIS and COLITIS+cur*Sm*SWP mice (Table 1).

**Table 1:** Cytokine profile in serum measured by CBA.

	CONTROL	COLITIS	COLITIS+cur*Sm*SWP	COLITIS+prev*Sm*SWP
**CBA serum (pg/ml)**				
IL-2	0.0±0.0	0.8±0.3#	0.5±0.2	0.3±0.1
IFN-γ	0.0±0.0	22.6±8.8	38.6±19.3#	17.5±6.9
TNF	3.0±0.6	83.6±12.5#	79.7±19.4#	75.0±11.5#
IL-6	0.0±0.0	8.1±1.8#	7.9±2.4#	4.5±1.0
IL-17A	1.4±0.7	6.1±0.8	4.8±1.5	14.7±5.8# **^,^** *
IL-4	0.0±0.0	0.0±0.0	0.6±0.6	0.0±0.0
IL-10	2.7±0.9	3.7±0.8	3.8±0.8	2.3±0.9

Data are presented as mean ± SEM. One-way ANOVA with LSD post-hoc test was used to compare the results between the CONTROL, COLITIS and COLITIS+cur*Sm*SWP groups or between CONTROL, COLITIS and COLITIS+prev*Sm*SWP groups.

# : *P*≤0.05, significantly different from CONTROL group;

*: *P*≤0.05, significant difference between COLITIS and COLITIS+prev*Sm*SWP groups. N = 9–12 per animal group. Abbreviations: CBA: cytometric bead array; cur: curative; IFN-γ: interferon-γ; IL: interleukin; prev: preventive; *Sm*SWP: *Schistosoma mansoni* soluble worm proteins; TNF: tumor necrosis factor.

### Effect of preventive *Sm*SWP treatment on the inflammatory parameters during colitis

**Figure 8:**
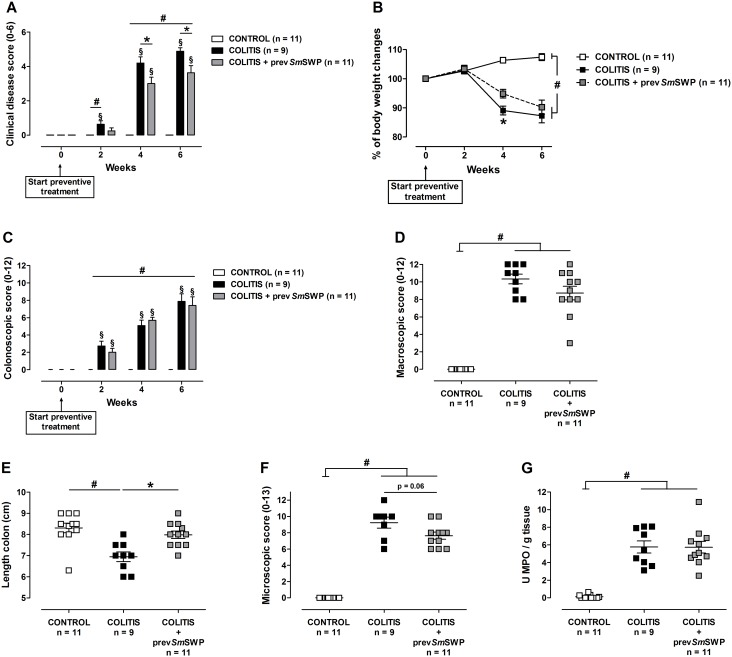
Effect of preventive treatment with *Sm*SWP on inflammatory parameters. Effect on clinical disease score (**A**), body weight (**B**), colonoscopic score (**C**), macroscopic inflammation score (**D**), colon length (**E**), microscopic inflammation score (**F**) and MPO activity (**G**). Data are presented as mean ± SEM. Generalized Estimations Equations was used to analyze the evolution of the body weight, the clinical disease score and colonoscopic score over time and an LSD post-hoc analysis was applied. One-way ANOVA with LSD post-hoc test was used to compare the results of macroscopic and microscopic scores, colon length and MPO activity between groups. ^#^: *P*≤0.05, significantly different from CONTROL group; *: *P*≤0.05, significant difference between the COLITIS and COLITIS+prev*Sm*SWP groups; ^§^: *P*≤0.05, significant increase in score over time within groups; “n” representing the number of mice. Abbreviations: LSD: least significant difference; MPO: myeloperoxidase; prev: preventive; SEM: standard error of the mean; *Sm*SWP: *Schistosoma mansoni* soluble worm proteins.

CONTROL mice showed no clinical or colonoscopic signs of colitis (Figure 8A–C), no macroscopic or microscopic damage and had low colonic MPO activity (Figure 8D–G).

The clinical disease score of COLITIS mice significantly increased over time (Figure 8A). Preventive administration of *Sm*SWP ameliorated the disease symptoms since the clinical disease score of COLITIS+prev*Sm*SWP mice remained significantly lower than that of COLITIS mice from week 4 onward (Figure 8A). Furthermore, COLITIS+prev*Sm*SWP mice showed a less severe body weight loss compared to COLITIS mice, as they lost respectively 9.5% and 13% of their initial body weight during the treatment period (Figure 8B).

Colonoscopic examination of the colon yielded no differences between COLITIS and COLITIS+prev*Sm*SWP mice and the signs of mucosal inflammation gradually increased over time in both groups as evidenced by significantly increased colonoscopic scores (Figure 8C).

The mucosal damage scored during the macroscopic examination was comparable between COLITIS and COLITIS+prev*Sm*SWP mice (Figure 8D). However, preventive *Sm*SWP treatment significantly increased colon length and tended to decrease (*P* = 0.06) the microscopic inflammation score of the COLITIS+prev*Sm*SWP mice compared with COLITIS mice (Figure 8E, F). Comparable MPO activities were seen between COLITIS and COLITIS+prev*Sm*SWP mice (Figure 8G).

### Effect of preventive *Sm*SWP treatment on the immunological response during colitis

**Figure 9:**
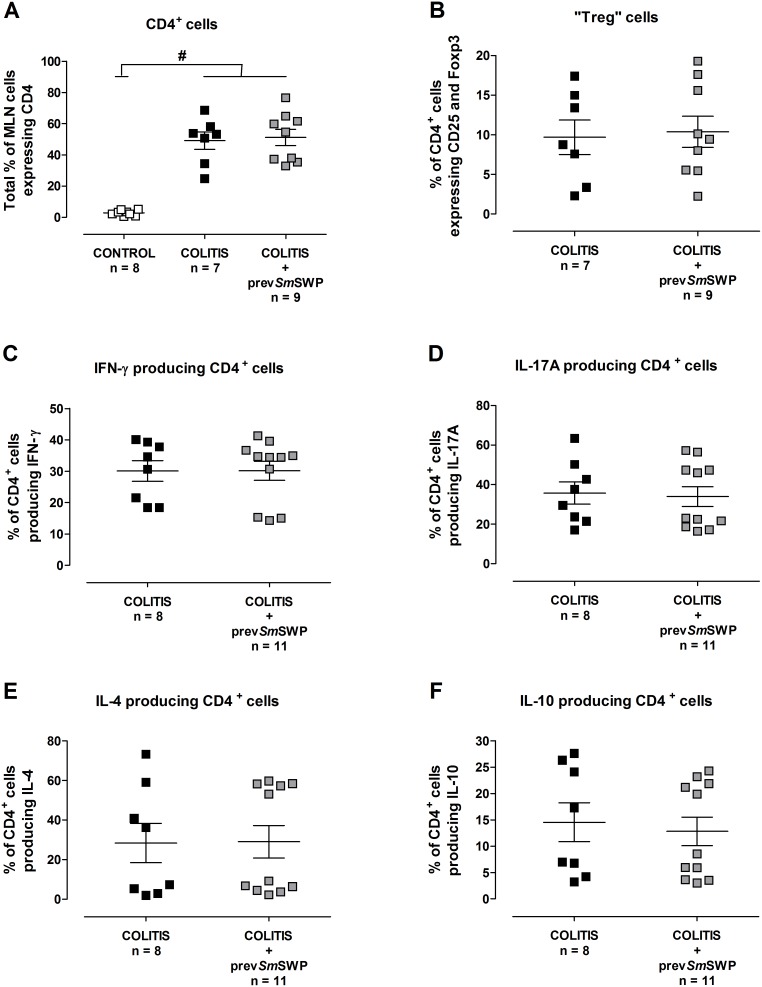
Flow cytometric T cell characterization of the MLN cells after preventive *Sm*SWP treatment. Total % of CD4^+^ cells (**A**), % of CD4^+^ cells expressing CD25 and Foxp3 (probably Treg cells) (**B**), % of CD4^+^ cells producing IFN-γ (**C**), % of CD4^+^ cells producing IL-17A (**D**), % of CD4^+^ cells producing IL-4 (**E**) and % of CD4^+^ cells producing IL-10 (**F**). Data are presented as mean ± SEM. One-way ANOVA with LSD post-hoc test or an unpaired Student’s *t* test was used as an appropriate approach to compare the flow cytometric results between groups. ^#^: *P*≤0.05, significantly different from CONTROL group; “n” representing the number of mice. Abbreviations: CD: cluster of differentiation; Foxp3: forkhead box p3; IFN-γ: interferon-γ; IL: interleukin; LSD: least significant difference; MLN: mesenteric lymph nodes; %: percentage; prev: preventive; SEM: standard error of the mean; *Sm*SWP: *Schistosoma mansoni* soluble worm proteins.

Flow cytometric T cell characterization of the MLN cells showed an equal upregulation of CD4^+^ cells in COLITIS and COLITIS+prev*Sm*SWP mice compared with CONTROL mice (Figure 9A). As expected, upregulation of CD4^+^ cells was absent in CONTROL mice (Figure 9A). Further analyses showed comparable percentages of CD4^+^ cells expressing CD25 and Foxp3 (probably Treg cells) and CD4^+^ cells producing IFN-γ, IL-17A, IL-4 and IL10 in COLITIS and COLITIS+prev*Sm*SWP mice (Figure 9B–F).

As shown in Figure 6D, preventive treatment with *Sm*SWP caused a significant rise in the colonic mRNA expression of IL-4 (a 2-fold increase) in COLITIS+prev*Sm*SWP mice compared with COLITIS mice, whereas no effect was seen on the expression of IL-10 mRNA. The expression of proinflammatory IL-17A and IFN-γ mRNA was downregulated in the colon after preventive *Sm*SWP treatment, but statistical significance was not reached (Figure 6D).

**Figure 10:**
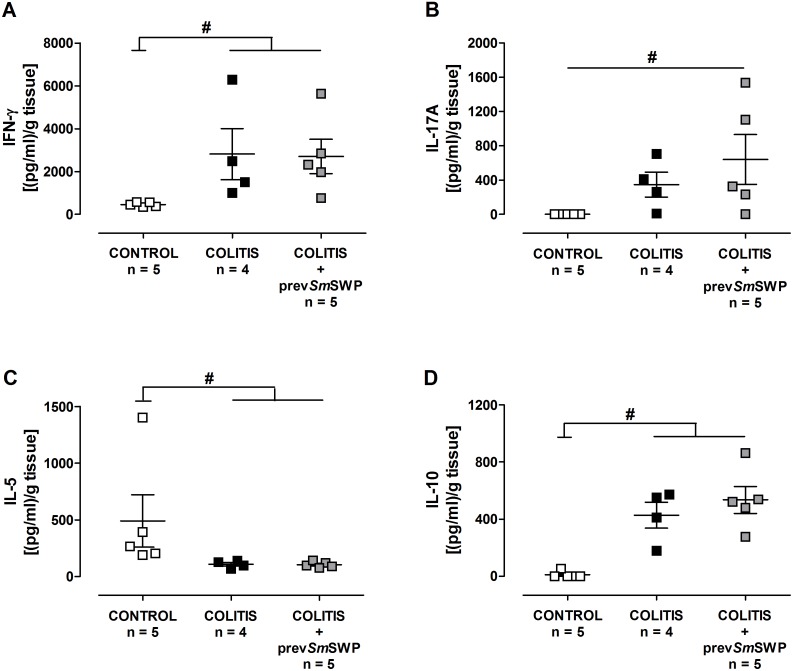
Effect of preventive treatment with *Sm*SWP on the cytokine profile in colon supernatants. Concentrations of IFN-γ (**A**), IL-17A (**B**), IL-5 (**C**) and IL-10 (**D**) were measured by ELISA in colon supernatants. Data are presented as mean ± SEM. One-way ANOVA with LSD post-hoc test was used to compare the concentrations between groups. ^#^: *P*0.05, significantly different from CONTROL group; “n” representing the number of mice. Abbreviations: ELISA: enzyme-linked immunosorbent assay; IFN-γ; interferon-γ, IL: interleukin; LSD: least significant difference; prev: preventive; SEM: standard error of the mean; *Sm*SWP: *Schistosoma mansoni* soluble worm proteins.

ELISA analysis did not reveal any differences in the concentrations of IFN-γ, IL-17A, IL-5 and IL-10 in colon supernatants between COLITIS and COLITIS+prev*Sm*SWP mice (Figure 10).

The serum cytokine concentrations, as detected via CBA analysis, were low. However, the preventive *Sm*SWP treatment resulted in a significant increase in IL-17A in the serum of COLITIS+prev*Sm*SWP mice compared with COLITIS mice. The serum concentrations of the other cytokines tested (IL-2, IFN-γ, TNF, IL-6, IL-4, IL-10) were comparable between COLITIS+prev*Sm*SWP and COLITIS mice (Table 1).

## Discussion

In this study, we provide evidence that proteins derived from adult *Schistosoma mansoni* worms (*Sm*SWP) have a beneficial effect on colitis on the long-term in the adoptive transfer mouse model, both in a curative and a preventive set-up. However, the therapeutic potential of the preventive *Sm*SWP treatment was less pronounced than that of the curative one.

In the CD4^+^CD25^−^CD62L^+^ T cell adoptive transfer colitis model, only non-activated non-Treg (CD25^−^) CD4^+^ T cells with migratory capacity (CD62L^+^) were injected in immunodeficient SCID mice, which lack functional T and B cells but have normal numbers of natural killer cells, macrophages, dendritic cells and granulocytes [33], [34]. This injection of naive CD4^+^CD25^−^CD62L^+^ T cells led to the development of colitis, which has been proven to result from a deficiency in Treg cells [16], [35]. This mouse model is considered a chronic colitis model, with a progressive inflammation eventually leading to death [36], [37].

Clinically, intestinal inflammation was monitored using different approaches. To evaluate the progression of clinical illness over time we used a clinical disease score, while colonoscopy was performed to monitor intestinal inflammation over time, without the need to sacrifice the animals [25]. Chronic colitis in our model was characterized by a disturbed architecture of the mucosa, infiltration of the lamina propria by inflammatory cells and destroyed crypts. In addition, colitis mice had a significantly shorter and narrower colon compared to controls. Colitis also significantly increased the colonoscopic, macroscopic and microscopic scores and the MPO activity. MPO activity is a measure of the number and activity of neutrophil granulocytes, one of the ‘first responder’ inflammatory cells to migrate towards the site of inflammation. Furthermore, a severe loss in body weight, a reduced physical activity and diarrhea were the apparent clinical features of this model.

Immunologically, experimental colitis was characterized by proinflammatory responses with upregulation of colonic IFN-γ and IL-17A levels, thus resembling the immune profile of Crohn’s disease. In detail, real-time PCR data from colonic tissues showed very high expression levels of proinflammatory IL-17A and IFN-γ mRNA in the colon of colitis mice, as well as an increase in colonic IL-4 and IL-10 mRNA, albeit to a lesser extent. On the protein level, ELISA analysis of colon supernatants demonstrated increased concentrations of IFN-γ, IL-17A and IL-10 in the colons of mice with colitis. In addition in MLN, the injected naive T cells mainly differentiated into CD4^+^ cells producing IFN-γ (Th1 cells), IL-17A (Th17 cells), IL-4 (Th2 cells) and IL-10 (Th2, Th9 or Treg cells) as shown by flow cytometry. Systemically, CBA analysis showed that the concentrations of IFN-γ and IL-17A were increased in the serum of mice with colitis.

Although the beneficial effect of helminth treatment on colitis is well-established, most animal studies have tested treatment with living helminth infections or helminth-derived molecules in a preventive set-up in acute animal models of colitis [11], [16]. From a clinical point of view, however, a curative protocol would be preferable. Therefore, in the present study we compared the effects of a preventive and a curative treatment protocol in a mouse model of chronic colitis. The dose of 25 µg *Sm*SWP in a total volume of 100 µl PBS was chosen based on previous research conducted in our lab by Ruyssers *et al.* where the maximal effect was reached with a dose of 25 µg *Sm*SWP and a higher dose did not further ameliorate the inflammatory parameters in an acute chemical colitis model [23]. The time schedule of *Sm*SWP administration was based on Zaccone *et al.* who showed that the *Schistosoma mansoni* soluble worm antigens could completely prevent the onset of type 1 diabetes in mice when treatments were started at 4 weeks of age [29].

Curative *Sm*SWP treatment induced a significant decrease in the clinical disease score, the colonoscopic score, the macroscopic and microscopic inflammation scores and MPO activity at week 6, whereas the colon length was significantly increased back to normal values indicating a beneficial effect on the colonic mucosal damage and the general sickness behavior. Body weight data were less conclusive, however we need to look critically at the body weight data as colitis mice were fed with pellets in their cages because of their illness while the other groups of mice were fed as usual with pellets on top of their cages. From these experiments we like to conclude that the previously shown beneficial effect of *Sm*SWP treatment in a model of acute TNBS colitis [23] was confirmed in a chronic colitis model supporting the concept of helminth soluble proteins as an interesting therapeutic strategy.

Limitations of the available *Sm*SWP mixtures prevented us from including a control group treated with *Sm*SWP, but we know from our previous experiments that *Sm*SWP treatment (25 µg) given 6 h after TNBS colitis induction, did not affect the inflammatory parameters studied in control Swiss OF1 mice (clinical disease score, macroscopic and microscopic inflammation scores and MPO activity) [23].

Preventive administration of *Sm*SWP caused a significant reduction in the clinical signs of illness from week 4 onward, led to a significant increase in colon length and revealed a tendency towards a reduced microscopic inflammation score. Again body weight data were less conclusive. Interestingly, MPO activity and mucosal damage (colonoscopically and macroscopically monitored) were not significantly affected by preventive *Sm*SWP treatment at week 6. Thus, in contrast to the curative treatment, a beneficial effect was not apparent in all inflammatory parameters. We can hypothesize that at the beginning of the preventive treatment protocol an insufficient number of T cells is available to exert their full therapeutic potential. However, Hang *et al.* showed that immunocompromised mice exposed to *Heligmosomoides polygyrus* helminth infection before transfer of IL10^−/−^ colitogenic T cells were protected from colitis as the interaction with innate immune cells (such as dendritic cells) was sufficient to provide initial protection against colitis thus not requiring T or B cells [38].

We further investigated the immunological effects underlying the beneficial effect of *Sm*SWP on experimental colitis. Interestingly, both the curative and the preventive *Sm*SWP treatment caused a downregulation of the mRNA expression of the proinflammatory cytokines IL-17A and IFN-γ and significantly upregulated IL-4 mRNA expression in the colon. IL-10 mRNA expression was not affected after *Sm*SWP treatment. Unfortunately, this colonic immunomodulatory effect of *Sm*SWP could not be confirmed at the protein level by ELISA analysis: no direct statistical significant differences were found between *Sm*SWP-treated and PBS-treated colitis mice but curative administration of *Sm*SWP did lower the concentrations of IFN-γ and IL-17A in the colon supernatants, resulting in a loss of significance between these *Sm*SWP-treated colitis and control mice. The use of colonic supernatants instead of colonic tissue might play a role in the differences between the ELISA data and real-time PCR data, as the use of supernatants presumes the secretion of cytokines before they can be detected in our set-up.

The flow cytometric T cell characterization in the MLN showed that neither the curative nor the preventive *Sm*SWP therapy had any effect on the number of CD4^+^ MLN cells expressing CD25 and Foxp3 (probably Treg cells), or on the number of CD4^+^ MLN cells producing IFN-γ (Th1 cells), IL-17A (Th17 cells), IL-4 (Th2 cells) or IL-10 (Th2, Th9 or Treg cells). Although *Sm*SWP treatment did not influence the number of the previous mentioned cell types in the MLN, it could be possible that *Sm*SWP affected the cell functions instead. However, we did not further investigate that. Another explanation one can think of could be that in response to the *Sm*SWP, differentiated T cells from the MLN were homing to the gut mucosa at the moment the flow cytometric T cell characterization was performed. CBA analysis in serum samples revealed no effect of curative *Sm*SWP treatment on the concentrations of IL-2, IL-4, IL-6, IFN-γ, TNF, IL-17A and IL-10, but showed a significant increase in IL-17A upon preventive *Sm*SWP treatment. In general, these immunological data indicate that *Sm*SWP are capable of restoring the disturbed intestinal immune balance and exert their protective effect locally in the colon, since the protective effect was primarily seen in colonic samples but not in MLN or serum. Our data derived from the analyses of colonic samples constitute a valuable addition to the existing scientific literature of helminth-based therapy and inflammatory bowel disease.

It is important to critically evaluate some methodological issues when interpreting our data. Although we aimed at defining the role of T-cell mediated responses induced by *Sm*SWP, real-time PCR was performed on whole-colonic tissue, indicating that the genes expressed could be derived from adaptive immune cells as well as from innate immune cells. The same is true for the interpretation of the CBA and ELISA data on serum and colonic supernatants. The majority of experimental studies investigating the underlying immunological mechanisms of helminthic therapy focused on T cell responses [11]. However recent studies dealing with the possible mechanisms of action of helminth-based therapy in IBD focus more on the innate immune cells, as these are the first cells to respond to helminths and are consequently able to affect cells of the adaptive immune system [11], [38]–[47].

Next, the exact composition of the *Sm*SWP mixture used in our experiments is unknown. Recently Boukli *et al.* identified several proteins in adult whole-worm extracts of *Schistosoma mansoni* including oxidative stress-related, energy metabolism-related, proteosomal and structural proteins [48]. Furthermore, Ferreira *et al.* showed that boiling products of *Ancylostoma caninum* resulted in an abrogation of their protective effects, indicating that the protection was entirely due to protein moieties [46]. Based on these findings we assume that our *Sm*SWP mixtures mainly consist of proteins. However, upon measuring the lipopolysaccharides (LPS) content in different *Sm*SWP mixtures, we found LPS to be present in a concentration range of 0.007 to 0.030 endotoxin units (EU)/µg protein (unpublished data). Although these concentrations can be regarded as low, they provide evidence that also non-protein components are present in our *Sm*SWP preparations.

Finally the dose and the time schedule of administration of worm protein mixtures could be investigated in more detail. Although our previous data suggest a narrow time window of the beneficial effect of the mixtures used in our protocols, different doses, time schedules and time of sacrifice need to be investigated in more detail, as well as the possibility of desensitization or tolerance after administration of multiple doses.

## Conclusion

Curative treatment of chronic colitis with *Sm*SWP reduced the severity of colitis induced by the adoptive transfer of CD4^+^CD25^−^CD62L^+^ T cells in immunocompromised SCID mice, as shown by a significant improvement of all inflammatory parameters studied, except one. The beneficial effect of preventive *Sm*SWP treatment on experimental colitis was less pronounced, since this beneficial effect was only reflected in some of the inflammatory parameters studied. Our results demonstrate that helminth antigen-induced amelioration of experimental colitis is associated with a downregulation of the proinflammatory cytokines IFN-γ and IL-17A and the upregulation of the anti-inflammatory cytokine IL-4 in the colon. These results hence lend further support to the use of helminth-derived therapy in IBD.
